# The Redox Sensor TXNL1 Plays a Regulatory Role in Fluid Phase Endocytosis

**DOI:** 10.1371/journal.pone.0001144

**Published:** 2007-11-07

**Authors:** Michela Felberbaum-Corti, Etienne Morel, Valeria Cavalli, Francis Vilbois, Jean Gruenberg

**Affiliations:** 1 Department of Biochemistry, University of Geneva, Geneva, Switzerland; 2 Serono Pharmaceutical Research Institute, Plan-les-Ouates, Geneva, Switzerland; University of California at Berkeley, United States of America

## Abstract

**Background:**

Small GTPases of the Rab family can cycle between a GTP- and a GDP-bound state and also between membrane and cytosol. The latter cycle is mediated by the Guanine Nucleotide Dissociation Inhibitor GDI, which can selectively extract GDP-bound Rab proteins from donor membranes, and then reload them on target membranes. In previous studies, we found that capture of the small GTPase Rab5, a key regulator of endocytic membrane traffic, by GDI is stimulated by oxidative stress via p38MAPK, resulting in increased fluid phase endocytosis.

**Methodology/Principal Findings:**

When purifying the GDI stimulating activity we found that that it copurified with a high MW protein complex, which included p38MAPK. Here we report the identification and characterization of another component of this complex as the thioredoxin-like protein TXNL1. Our observations indicate that TXNL1 play a selective role in the regulation of fluid phase endocytosis, by controlling GDI capacity to capture Rab5.

**Conclusions/Significance:**

Oxidants, which are known to cause cellular damage, can also trigger signaling pathways, in particular via members of the thioredoxin family. We propose that TXNL1 acts as an effector of oxidants or a redox sensor by converting redox changes into changes of GDI capacity to capture Rab5, which in turn modulates fluid phase endocytosis.

## Introduction

Cellular uptake of particles, solutes, lipids, and membrane proteins, including receptor–ligand complexes is mediated by clathrin-dependent endocytosis, macropinocytosis and also by other pathways, in particular rafts and caveolae [1], [2]. Molecules internalized by the clathrin pathway, and by at least some of the other routes, are delivered to early endosomes, where sorting occurs. Some molecules are then recycled back to the plasma membranes, e.g. housekeeping receptors, or transported to the trans-Golgi network, while others, like the downregulated signaling receptors, are transported to late endosomes and then lysosomes for degradation [3]. Compelling evidence now shows that signaling pathways control many endocytic transport steps [4], [5], [6]. In particular, a genome-wide analysis has revealed that an unexpectedly high number of kinases are implicated in clathrin and caveolar/raft endocytosis [5]. Further, many of these “endocytic” kinases also function in various signaling pathways, strengthening the notion that endocytosis and signaling are tightly coupled [5].

The small GTPase Rab5 plays a crucial role in the cross talk between endocytic membrane traffic and signaling. This GTPase regulates early endocytic events [7], and coordinates entry into the cells by endocytosis and macropinocytosis, in particular via its effector Rabankyrin-5 [8]. Like other GTPases, Rab5 cycles between GTP- and GDP-bound states, and, interacts in the active GTP-bound state with its effectors, which mediates Rab5-dependent functions. In addition, GTPases of the Rab family can also cycle between membrane and cytosol via the Guanine nucleotide Dissociation Inhibitor (GDI). GDI has the capacity to extract Rab proteins in their inactive GDP-bound state from cellular membranes, and then to deliver them to their appropriate target membranes. We previously found that p38MAPK phosphorylates GDI and thereby increases GDI capacity to capture early endosomal Rab5, which in turn stimulates endocytosis [9], [10], presumably by increasing Rab5 cycling rates. Similarly, long term depression (LTD) triggers p38MAPK activation and leads to AMPA receptor endocytosis, probably by stimulating GDI∶Rab5 complex formation [11] or by directly activating Rab5 at the plasma membrane [12]. p38MAPK activation is also required and sufficient for endocytosis of the µ-opioid receptor [13] and the epidermal growth factor receptor (EGFR) [14], [15], via phosphorylation of the Rab5 effector EEA1 [13] and EGFR [15], respectively. Consistently, silencing several kinases involved in clathrin endocytosis induces p38MAPK phosphorylation and its association with endosome-like structures [5].

Here, we report that the thioredoxin-like protein TXNL1 modulates GDI functions in the cycle of Rab5 and regulates fluid phase endocytosis. At steady state (under cytosolic reducing conditions), TXNL1 is found in the cytosol partially associated both with p38MAPK and with GDI. A fraction of TXNL1 and p38MAPK is also associated with early endosomes, where TXNL1 may interfere with the capture of Rab5 by GDI.

## Results

### p38MAPK and TXNL1

**Figure 1:**
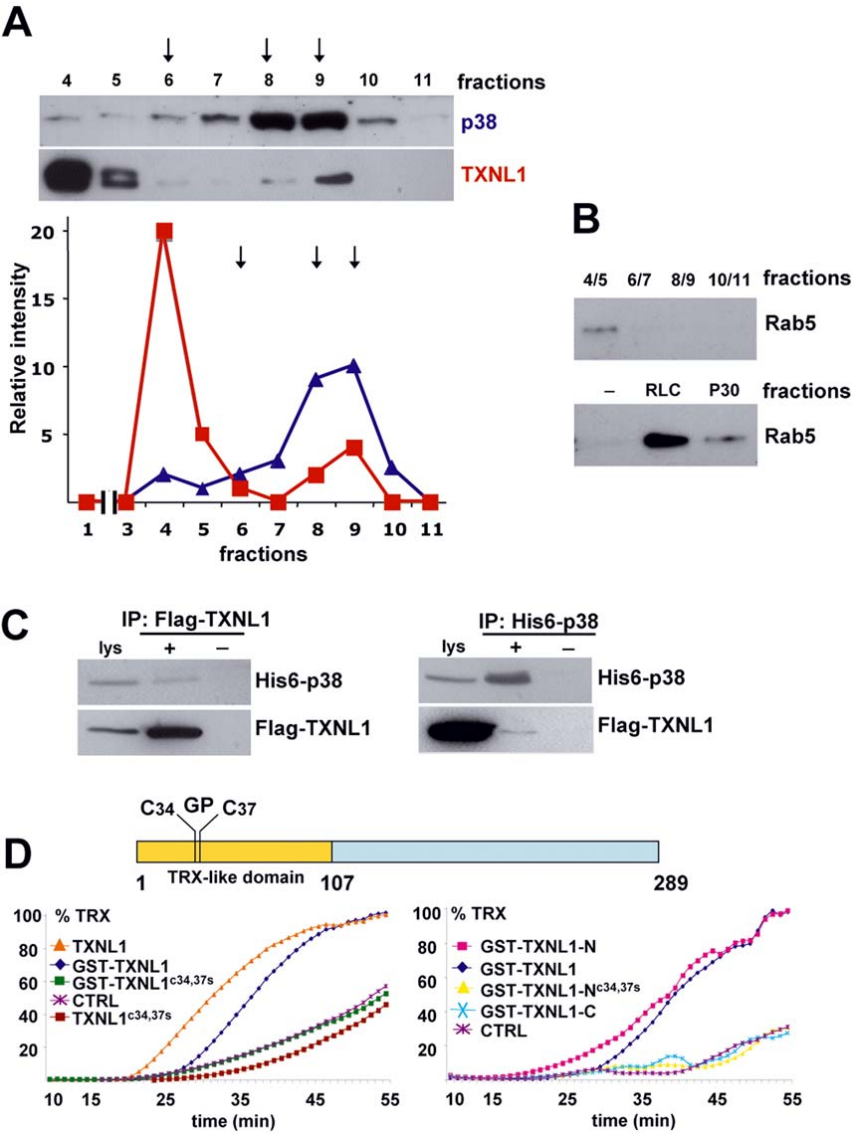
TXNL1 and p38MAPK. (A) Rat liver cytosol (RLC, 30 mg) was precipitated in 30% (NH_4_)_2_SO_4_ (P30). The precipitate was fractionated by gel filtration chromatography on a Superose 12 column, and fractions analyzed by western blotting. The lower panel shows the densitometric scan of the gels in the upper panel (red squares: TXNL1; blue triangles: p38MAPK). Arrows point at calibration markers: ferritin 440 kDa; aldolase 156 kDa; albumin 67 kDa. The bulk of GDI is associated with Rab proteins [10] and is thus present as complexes of ≈80 kD [22], which elute in fractions 8-9 on the Superose 12 column. (B) The Superose 12 fractions (A) were pooled 2 by 2 and assayed for their capacity to stimulate 1 µM GST-GDI to extract early endosomal Rab5 [39]. After endosome removal on gradients, GST-GDI with bound Rab5 was retrieved onto glutathione beads and analyzed by western blotting. For comparison, the activity of 100 µg RLC or P30 is shown. The fractionation (A) and analysis (B) was repeated 3 times, and representative examples are shown. (C) HeLa cells were co-transfected with Flag-TXNL1 and His6-p38MAPK. Immunoprecipitation (IP) from lysates (lys: 5% of total) was with (+) or without (−) the indicated antibodies, and analysis was by western blotting. Each experiment was repeated at least 3 times, and (C) shows a representative example. (D) The outline of TXNL1 is shown with the CPGC motif in the TRX-like N-terminal domain (residues 1–107). Purified recombinant proteins were assayed (5 µM) before or after GST cleavage for their capacity to reduce insulin disulfide bonds in the presence of dithiothreitol, using dithiothreitol alone as a control (CTRL). Data are normalized to values obtained with *E.Coli* thioredoxin (TRX).

We previously reported that a cytosolic activity stimulates the capacity of GDI to capture and extract Rab5 from early endosomal membranes [9]. This activity eluted at a high (≈400 kDa) apparent MW by gel filtration (Fig 1 A–B and see below) and was recovered following purification, in a fraction containing 5 major polypeptides, including p38MAPK [9]. Since p38MAPK alone could activate GDI [9], polypeptides co-purifying with p38MAPK presumably fulfill regulatory functions. Amongst these, we identified by tandem mass spectrometry TXNL1 or TRP32 (thioredoxin-related protein of 32 kDa) [16], [17]—a protein that had been previously implicated in the cellular response to sugar starvation stress [18]. TXNL1 is an ubiquitous protein with a N-terminal thioredoxin-like domain [19] and a C-domain of unknown functions (see Fig 1D). When ectopically expressed, FLAG-tagged TXNL1 co-immunoprecipitated His6-p38 and vice versa, but with low efficiency (Fig 1C). This suggests that a minor fraction only of these proteins formed a presumably transient complex—endogenous TXNL1 could not be immunoprecipitated with our antibodies. Importantly, however, after gel filtration of rat liver cytosol, the bulk of endogenous TXNL1 was recovered in high MW fractions (Fig 1A fractions 4-5, red symbols), well separated from the position of the TXNL1 monomer (Fig 1A, fraction 8-9). In fractions 4-5, the high MW form of TXNL1 co-purified with a portion of endogenous p38MAPK (Fig 1A fractions 4-5, blue symbols) and with the GDI stimulating activity (Fig 1B, fractions 4-5). The bulk of p38MAPK eluted at the position of the monomer (Fig 1A, fractions 8-9), as expected since p38MAPK is involved in many cellular responses and only a minor fraction of total p38MAPK may be involved in this pathway. In addition, p38MAPK association with the complex may be relatively labile. In any case, this suggests that TXNL1 is part of a protein complex that contains p38MAPK and exhibits GDI stimulating activity.

### TXNL1 redox activity

Since TXNL1 contains an N-terminal thioredoxin-like domain, including the conserved CGPC (Fig 1D) catalytic site, we investigated whether the purified recombinant protein could reduce the disulfide bonds of insulin in the presence of dithiothreitol, hence whether it was catalytically active. We also used a truncated version of TXNL1 corresponding to the N-terminal domain containing the thioredoxin-like domain (GST-TXNL1-N, residues 1–122), as well as the C-terminal domain alone (GST-TXNL1-C, residues 105–289). Both full length GST-TXNL1 and TXLN1 (after GST cleavage) exhibited reducing activity in vitro when compared to thioredoxin (TRX), as did the N-domain of TXNL1 (GST-TXNL1-N), but not the C-domain of TXNL1 (GST-TXNL1-C), as expected [19] (Fig 1D). Moreover, mutation of the two conserved cysteine residues in the CGPC motif to serines (C34,37S) abolished GST-TXNL1 and GST-TXNL1-N ability to reduce insulin (Fig 1D), indicating that the N-terminal thioredoxin-like domain of TXNL1 exhibits catalytic activity, and is thus functionally related to thioredoxin.

### TXNL1 and phospho-p38MAPK are partially associated with early endosomes

**Figure 2:**
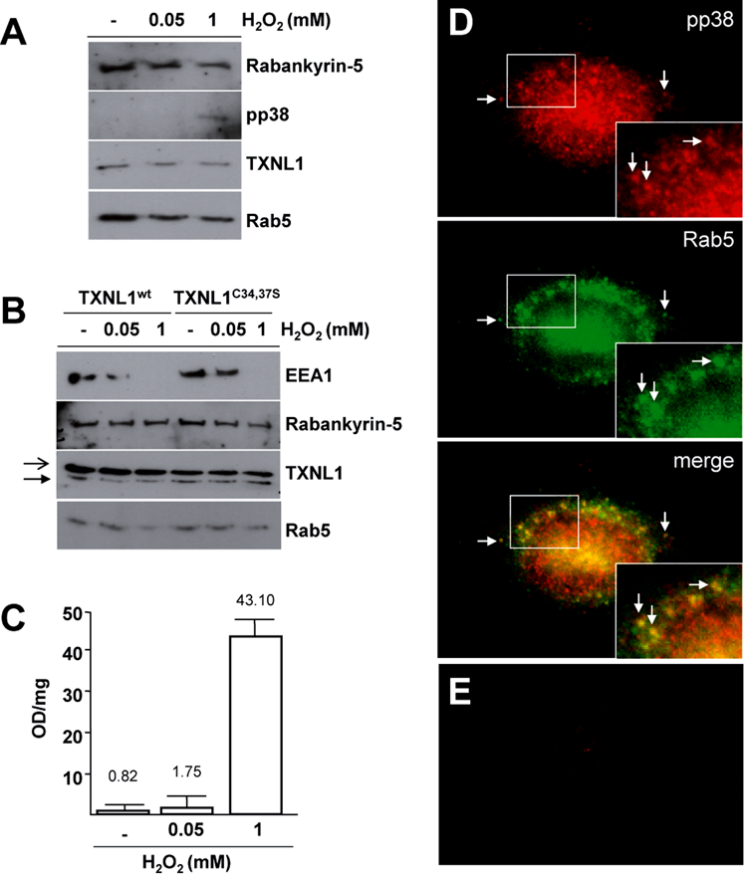
Association with early endosomes. (A) Early endosomes were prepared from BHK cells treated or not for 10 min with 0.05 or 1 mM H_2_O_2_ [39], and analyzed by western blotting. (B) Experiments were as in (A), but cells were transfected with Flag-TXNL1 or Flag-TXNL1^C34,37S^. Open and solid arrows point at Flag-tagged and endogenous TXNL1, respectively. Each experiment in (C) and (D) was repeated at least 5 times, and representative examples are shown. (C) Phospho-p38MAPK present in early endosomal fractions was quantified after lysis in 1% TX100 by sandwich ELISA (expressed as OD units per mg protein). The figure shows the mean of 3 experiments. Error bars represent standard deviations; the observed differences are significant according to Student's *t* test with p<0.0001 at each H_2_O_2_ concentration. (D) HeLa cells were analyzed by immunofluorescence using antibodies against phospho-p38MAPK (pp38) and Rab5, followed by labeled secondary antibodies. Arrows point at examples of endosomes containing both Rab5 and pp38 (see inset). A fraction of pp38MAPK colocalized with Rab5 on early endosomes, but we were unable to unambiguously localize pp38MAPK on the membrane of other organelles using various markers, presumably because detection was limiting. (E) Cells were processed as in (D), except that primary antibodies against phospho-p38MAPK were omitted, and then analyzed in the red channel.

TXNL1 is primarily a cytosolic protein [16], but small amounts (<5% total) co-sedimented with membranes (not shown). After floatation in gradients, the endogenous form of membrane-associated TXNL1 co-purified with Rab5 and its effectors Rabankyrin-5 (Fig 2A) and EEA1 (Fig 2B, solid arrow) in early endosomal fractions, as did the ectopically expressed FLAG-tagged form (Fig 2B, open arrow). Similarly, a minor fraction of phospho-p38MAPK could be detected on Rab5-containing early endosomes at steady state by immunofluorescence (Fig 2D)-but not by western blotting when detection was limiting (Fig 2A). Detection of p38MAPK was specific, since the signal was not observed when the primary antibody was omitted (Fig 2E).

We previously showed that oxidative stress partially releases Rab5 (bound to GDI) from endosomes, and that this, in turn, causes the dissociation of EEA1 ([9] and Fig 2B). When activated, phosphorylated p38MAPK becomes mainly translocated to the nucleus (not shown; see also [9] and references therein) but interestingly we found that amounts of endosome-associated phospho-p38MAPK also increased significantly after oxidative stress (Fig 2A and 2C). Under stress conditions, a temporal sequence may thus control the transient action of active p38 on GDI∶Rab5 before transit to the nucleus. In contrast, endosomal association of endogenous and FLAG-tagged TXNL1 was independent of the stress response (Fig 2A and 2B). Moreover, mutation of the two conserved cysteines in the CGPC catalytic site of the thioredoxin domain (TXNL1^C34,37S^) inhibited TXNL1 reducing activity (Fig 1D), but did not affect TXNL1 endosome association, whether or not cells were submitted to oxidative stress (Fig 2B).

### Rab5 capture by GDI is modulated by TXNL1

**Figure 3:**
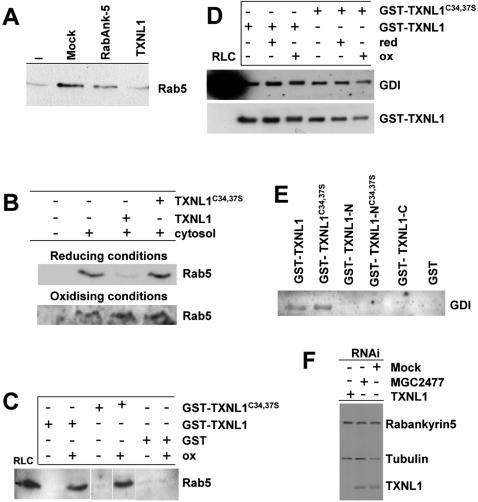
Rab5 capture by GDI is modulated by TXNL1. (A) The capture of early endosomal Rab5 by GDI was analyzed. In the assay, GST-GDI was incubated with early endosomes in the absence (−) or presence of cytosol prepared from mock-transfected cells (mock), or from cells overexpressing either the Rab5 effector Rabankyrin-5 (RabAnk-5) or TXNL1. Rab5 bound to GDI was then retrieved and analyzed as in Fig 1B. The experiment was repeated 3 times, and a representative example is shown. (B) The capture of early endosomal Rab5 by GDI was analyzed as in (A), except that the assay was in the absence (−) or presence (+) of rat liver cytosol and 500 nM recombinant TXNL1 or TXNL1^C34, 37S^ (after GST removal). Recombinant proteins were pre-reduced for 30 min with 1 mM DTT on ice (reducing conditions), or pre-oxidized for 15 min with 10 mM H_2_O_2_ at 25°C (oxidizing conditions). In both cases, proteins were then diluted 12 fold in the reaction mixture and the same final DTT or H_2_O_2_ concentrations were used when TXNL1 or TXNL1^C34, 37^ were omitted. These experiments were repeated 5 times, and representative examples are shown (C) Pulldown of rat liver cytosol (RLC) using purified GST fusion proteins that were (+) or not (−) pre-oxidized (Ox) as in (B). GST-proteins were retrieved onto immobilized beads and analyzed by western blotting with anti-Rab5 antibodies (all lanes are from the same gel). These experiments were repeated 4 times, and representative examples are shown. All lanes in the panel are from the same experiment. (D) Pulldown of rat liver cytosol (RLC) using purified GST-fusion proteins that were (+) or not (−) pre-reduced (Red) or pre-oxidized (Ox) as in (B). Analysis was by western blotting with anti-GST or anti-GDI antibodies. These experiments were repeated 3 times, and representative examples are shown (E) Pulldowns with GST-fusion proteins (C, and see Fig 1D) were analyzed by western blotting with anti-GDI antibodies. These experiments were repeated 3 times, and representative examples are shown (F) HeLa cells were either mock transfected or transfected with siRNA duplexes for 72 h (MGC2477 hypothetical protein as RNAi control). Total cell-extracts were analyzed by western blotting with the indicated antibodies.

We then investigated whether TXNL1 modulated the capacity of GDI to extract Rab5 from purified early endosomes. We found that formation of the Rab5∶GDI complex was inhibited by cytosol prepared from cells overexpressing TXNL1, but not by cytosol from mock-transfected cells or from cells overexpressing the Rab5 effector Rabankyrin-5 (Fig 3A). This TXNL1-dependent inhibition was only observed with freshly prepared cytosol presumably because TXNL1 became oxidized during storage—a process that would not occur *in vivo* in the reducing environment of the cytosol. We thus tested whether the regulatory function of TXNL1 depended on its redox state. While the addition of the pre-reduced form of recombinant TXNL1 to control cytosol inhibited Rab5 extraction, the pre-oxidized form did not interfere with GDI activity (Fig 3B). Neither did the recombinant catalytically inactive TXNL1^C34,37S^ mutant Fig 3B), which presumably mimics a constitutively oxidized state [20]. We could not determine whether TXNL1 also regulates the capacity of GDI to capture other Rab proteins, because the detection was limiting with the antibodies against other Rab proteins that we had available. Clearly, it will be important to determine whether the same mechanism only controls endocytosis via Rab5, or controls selective transport steps via a sub-set of Rabs, or whether this mechanism operates in a more general manner in membrane traffic regulation. Our observations thus indicate that, in its reduced form, TXNL1 negatively regulates the activity of GDI to capture Rab5 *in vitro*, and suggest that TXNL1 regulatory functions depend on its catalytic activity hence its capacity to cycle between oxidized and reduced states.

### TXNL1 interactions with GDI∶Rab5 and GDI

Our observations that TXNL1 regulates GDI activity suggest that TXNL1 interacts with GDI and perhaps with the GDI∶Rab5 complex. Indeed, all Rab5 is bound to GDI in the cytosol [10], while only a minor fraction of GDI is complexed to Rab5 (GDI is an abundant protein). We thus tested whether, cytosolic Rab5 (bound to GDI) co-purified after pulldown with WT GST-TXNL1 or with the GST-TXNL1^C34,37S^ mutant, which presumably mimics a constitutively oxidized state. Surprisingly, Rab5 co-purified with both WT and mutant forms, but only in the presence of H_2_O_2_ (Fig 3C). This was not due to artificial cross-linking via *trans* disulfide bridges, since Rab5 mobility was unaffected in non-reducing gels (not shown). Neither were these interactions due to differences in the amounts of GDI pulled down by WT GST-TXNL1 or GST-TXNL1^C34, 37S^ (Fig 3D). Indeed, TXNL1 pulled down GDI equally well under reducing or oxidizing conditions—as did TXNL1^C34, 37S^ (Fig 3D), ruling out the possibility that TXNL1 was artificially cross-linked to GDI *in vitro*. Hence, TXNL1 independently of its redox state seemed to have the capacity to interact directly or indirectly with cytosolic GDI—as it did with endosomal membranes (Fig 2B). This interaction required full-length TXNL1, since neither the catalytic active N-domain (Fig 1D) nor the C-domain alone pulled down GDI (Fig 3E). We thus conclude that, while TXNL1 can interact with GDI independently of its redox state (Fig 3D), peroxide triggers or stabilizes direct or indirect interactions with the GDI∶Rab5 complex (Fig 3C). Oxidative conditions may thus facilitate TXNL1 interactions with the cytosolic GDI∶Rab5 complex (Fig 3C), concomitantly with increased Rab5 capture by GDI [9] and decreased GDI membrane-association [21].

It should be noted that in our gel filtration analysis (Fig 1A) we used a Superose 12 column, which does not provide sufficient resolution in the low MW range to separate GDI associated with Rab proteins [10] as complexes of ≈80 kD [22] from a putative complex of GDI∶Rab5 with TXNL1. Moreover, this complex should only correspond to a small fraction of total GDI, since GDI interacts with most Rab proteins [7].

### TXNL1 regulates endocytosis

**Figure 4:**
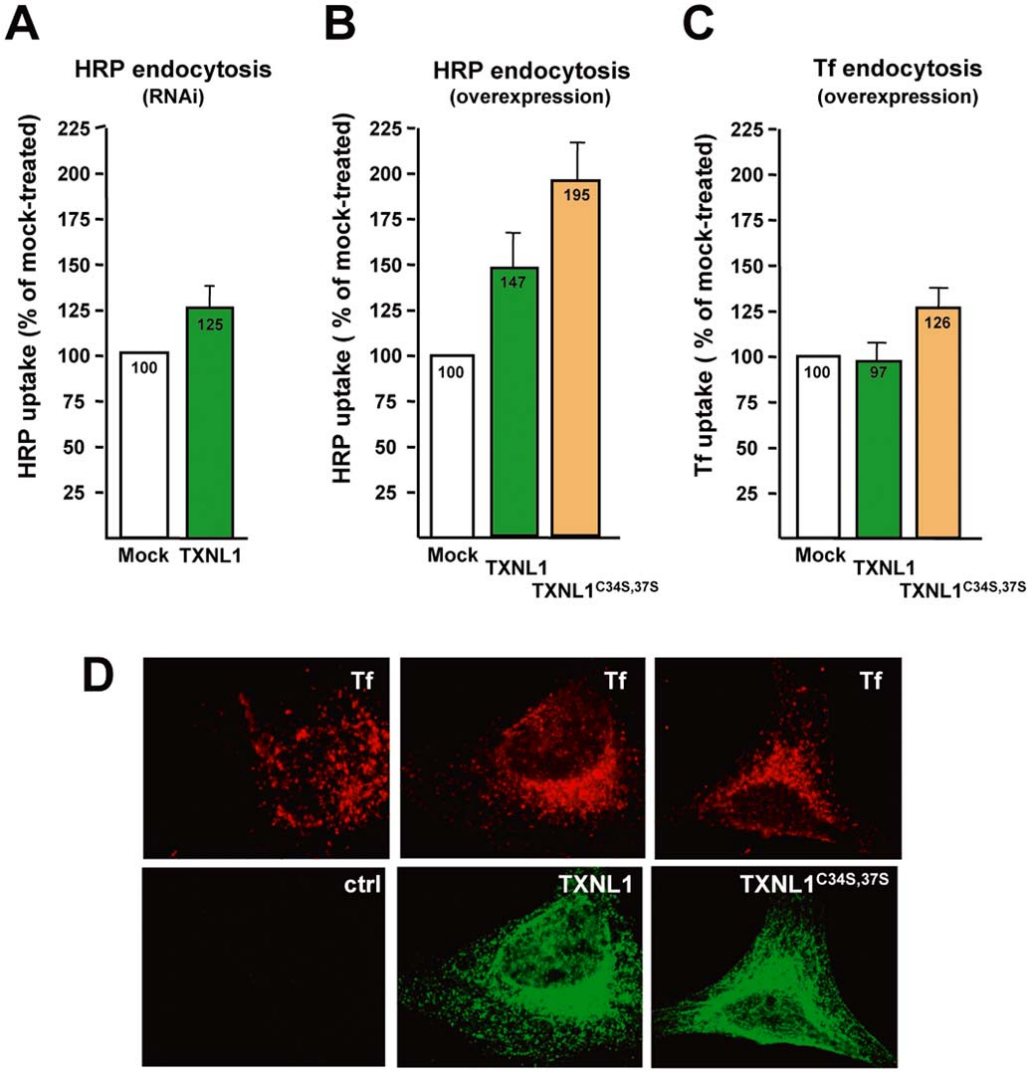
TXNL1 regulates fluid phase endocytosis. (A) HRP uptake for 5 min at 37°C was quantified in HeLa cells either mock-transfected or transfected with TXNL1-siRNA duplexes. Data are normalized to the uptake in mock-treated cells and are representative of at least three independent experiments (each in duplicate). The error bar represents the standard deviation; the observed differences are significant according to Student's *t* test with p<0.005 (B) HeLa cells were either mock transfected or transfected with Flag-TXNL1 or Flag-TXNL1^C34, 37S^. Cells were incubated with HRP for 5 min at 37°C and analyzed as in (A). The figure shows the mean of 3 independent experiments. Error bars represent the standard deviation; the observed differences are significant according to Student's *t* test with p<0.0001 under each condition (C–D) HeLa cells were either mock transfected or transfected with Flag-TXNL1 or Flag-TXNL1^C34, 37S^, as in (B), incubated with rhodamine-labeled transferrin (Tf), processed for immunofluorescence using anti-flag antibodies and visualized by double-channel fluorescence microscopy (D). The number of transferrin-containing endosomes was counted in 20 cells of duplicate samples in 3 independent experiments (as in D) and the quantification is shown in (C). Error bars represent the standard deviation; the observed differences after TXNL1^C34, 37S^ overexpression are significant (p<0.01), but not after TXNL1 overexpression (p<0.2).

Since TXNL1 appears to regulate Rab5 capture by GDI and to interact with GDI∶Rab5, we investigated TXNL1 potential role during endocytosis *in vivo*. To this end, uptake of the fluid phase tracer horseradish peroxidase (HRP) was quantified over short time-periods to ensure that only internalization was being monitored, rather than more distal transport steps. Knockdown of TXNL1 with dicer-generated siRNA duplexes (Fig 3F) did not interfere with HRP uptake, except perhaps by causing a small, marginal increase (Fig 4A) in line with the inhibitory effect of reduced TXNL1 in vitro (Fig 3B). Strikingly, overexpression of TXNL1^C34,37S^—the catalytically inactive mutant that presumably mimics the oxidized state—caused a marked (≈200%) increase in endocytosis (Fig 4B), consistently with observations that oxidative conditions accelerate endocytosis and facilitate Rab5 capture by GDI [9] as well as TXNL1 interactions with the GDI∶Rab5 complex. Some stimulation was also observed with WT TXNL1 (overexpressed to the same levels as TXNL1^C34,37S^, Fig 2B), presumably reflecting TXNL1 capacity to cycle from reduced to oxidized states—indeed, the constitutively oxidized mutant increased endocytosis in vivo, while reduced TXNL1 was inhibitory in vitro (Fig 3B). Consistently, TXNL1 overexpression did not cause Rab5 release from membranes (not shown), hence did not lead to GDI activation, demonstrating that TXNL1 levels are not limiting. By contrast, overexpression of TXNL1 or TXNL1^C34,37S^ did not stimulate clathrin-mediated endocytosis of the transferrin receptor to any significant extent (Fig 4D)—a marginal increase was observed with the TXNL1^C34,37S^ mutant (Fig 4C). These observations suggest that the massive increase in HRP endocytosis after TXNL1^C34,37S^ or WT TXNL1 overexpression occurred primarily via a clathrin-independent pathway, presumably macropinocytosis. Indeed, this pathway, which accounts for the uptake of large volumes of fluid, can be transiently up-regulated [1] and is under the direct control of Rab5 [8]. TXNL1 thus appears to serve as a redox sensor that selectively controls fluid phase endocytosis via GDI functions in the membrane-cytosol cycle of Rab5.

## Discussion

**Figure 5:**
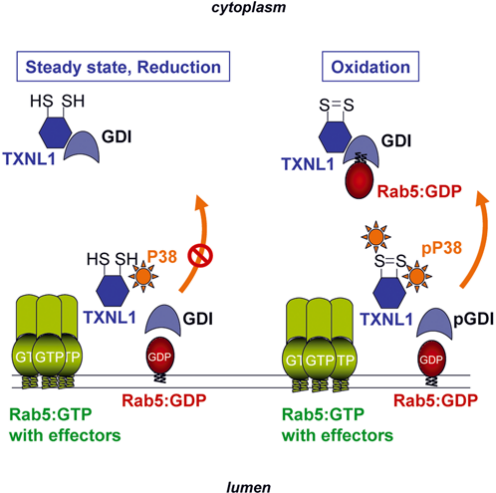
The TXNL1 cycle. The model outlines the proposed mechanism by which TXNL1 may regulate fluid phase endocytosis, by modulating GDI function in the membrane-cytosol cycle of Rab5. See text for further explanations. P38, p38MAPK; pP38, phosphorylated p38MAPK; GDI, Guanine nucleotide Dissociation Inhibitor; pGDI; phosphorylated GDI; Rab5∶GTP or GDP, Rab5 in the GTP- or GDP-bound state, respectively.

Previous studies have shown that p38MAPK regulates endoyctic membrane traffic by regulating GDI [9] and EEA1 [13] activity. In particular, we had found that p38MAPK increases the capacity of GDI to capture early endosomal Rab5 and stimulates endocytosis, presumably through a net increase in Rab5 cycling [9]. Here, we report that the thioredoxin-like protein TXNL1, which can be associated with p38MAPK, modulates GDI functions in the cycle of Rab5 and regulates fluid phase endocytosis. Our observations suggest that TXNL1 functions as an effector of oxidants or redox sensor that couples oxidative stress to endocytosis, by converting redox changes into a specific GDI∶Rab5-mediated endocytic response (see outline, Fig 5).

### Thioredoxins and the endocytic pathway

Previous studies have linked thioredoxin to the regulation of membrane traffic in yeast. Vacuole homotypic fusion [23], [24] and the fusion of ER-derived vesicles with Golgi membranes [25] depend on the LMA1 (low molecular weight activity 1) complex composed of thioredoxin and the cytosolic proteinase B inhibitor I^B^_2_. Thioredoxin catalytic activity appears to be dispensable for this role in membrane fusion [26]. Since we find that TXNL1 redox activity is required, TXNL1 function appears distinct from that of thioredoxin in fusion. Our findings rather support the view that TXNL1 acts as a physiological regulator of GDI∶Rab5-mediated endocytic response. Our observations also suggest that TXNL1 controls some, but not all, Rab5 functions, since TXNL1 primarily regulates fluid phase uptake, while Rab5 plays a dual role in endocytosis and macropinocytosis [8].

It is tempting to speculate that TXNL1 selectively regulates Rab5 cycling to and from specific endosomal effector platforms, presumably containing Rabankyrin-5 [8], involved in macropinocytosis regulation (see outline Fig 5). In the GTP-bound state, Rab5 can recruit its effectors (see outline Fig 5), and after GTP hydrolysis, inactive GDP-bound Rab5 is extracted by GDI [7], We previously reported that this process is stimulated by the action of p38MAPK [9]. We propose that, at steady state, i.e. under cytosolic reducing conditions, TXNL1 is found in the cytosol partially associated both with p38MAPK and with GDI, while a fraction of TXNL1 and p38MAPK are also associated with endosomal membranes. There, TXNL1 may interfere with GDI-mediated Rab5 extraction. This inhibitory function may be released by intracellular redox changes and TXNL1 oxidation, leading to increased activation of GDI on membranes via p38MAPK [9]. We thus conclude that TXNL1 functions as an effector of oxidants or a redox sensor that selectively couples oxidative stress and redox changes into GDI∶Rab5-mediated response in fluid phase endocytosis. This mechanism may contribute to control the turnover of membrane components for repair or degradation after stress.

### Thioredoxins as ROS effectors

Disturbed intracellular redox balance, also known as “oxidative stress”, is characterized by increased intracellular concentrations of highly reactive, oxidizing species, known as ROS (reactive oxygen species) and RNS (reactive nitrogen species). Cells can intentionally produce ROS during host defense and inflammatory response. However, in many instances ROS production may also fulfill signaling purposes [27], [28]. For instance, ROS-induced cystein oxidation is now considered a reversible and rapid modification that regulates protein functions [27], [29]. Further, oxidant species modulate the activity of two antioxidant proteins, namely peroxiredoxin [30] [31] and thioredoxin [32], [33], suggesting the existence of a dynamic crosstalk between ROS and ROS-scavenging system.

Thioredoxins (Trx) are the major dithiol reductants present in the cytosol [34] and act mainly as antioxidants by protecting cytosolic proteins from ROS-induced oxidative damages [35] and by directly scavenging ROS. However, thioredoxins, which are highly susceptible to ROS-induced oxidation, may also play an important role as effectors of oxidants. For instance, thioredoxin1 has redox-dependent inhibitory functions on ASK1 [36], a MAPKKK that activates JNK and p38MAPK and mediates stress-induced apoptosis. Similarly, the redox-sensitive interaction of nucleoredoxin—a thioredoxin-like protein with reducing activity—with Dishevelled probably regulates ROS-induced Wnt-ß-catenin signaling [37]. Finally, the thioredoxin-like protein PICOT/TXNL2 (protein kinase C-interacting cousin of thioredoxin), which presumably lacks enzymatic activity, interacts with protein kinase C-theta (PKCθ) inhibiting its activation of JNK/AP1 and NF-kB, probably by competing with thioredoxin or thioredoxin-reductase for substrate binding [38]. Future work will be needed to determine whether PICOT and TXNL1, which share a similar domain organization and mass, act as selective effectors of the stress pathways regulated by JNK and p38MAPK, respectively, although PICOT carries critical mutations in the thioredoxin-like active site. These observations highlight possible general functions of thioredoxin family members as redox sensors or regulators in signaling pathways that couple oxidative stress to cellular responses.

## Materials and Methods

### Cells, antibodies and reagents

Baby Hamster Kidney cells (BHK21) and HeLa cells were grown as described [9], [39]. Monoclonal antibodies were obtained from: His6 from Roche Diagnostics (Basel, CH); GST from Santa-Cruz Biotechnology, Inc. (San Diego, CA); TXNL1 from Abcam (Cambridge, UK); Rab5 and GDI from Reinhard Jahn (Göttingen, Germany); FlagM2 from Sigma Chemical Co. (St. Louis, MO); polyclonal antibodies against phospho-p38 and p38 were from Cell Signaling (Beverly, MA); fluorescently labeled secondary antibodies were from Jackson Immunoresearch Laboratories (West Grove, PA). The peptide KCSTKEIPIIKFDLNK, corresponding to a conserved C-terminal region of Rabankyrin-5 (amino acids 1136–1151) was used to raise a polyclonal antibody (Ab 16KK) in rabbits (CovalAb, Oullins Cedex, France). Glycerol 2-phosphate, Sodium Orthovanadate, ATP, GDP, hydrogen peroxide, horseradish peroxidase (HRP), *o*-dianisidine, recombinant *E. Coli* thioredoxin and bovine insulin were from Sigma Chemical Co. (St. Louis, MO); glutathione-Sepharose 4B beads, Benzamidine, Sepharose 4 fast flow, Factor Xa from Amersham (UK); transferrin-rhodamine from Molecular Probes (Eugene, OR); okadaic acid from Calbiochem (San Diego, CA); FuGene6 from Roche Diagnostics (Basel, CH). Oligonucleotides were synthesized by Eurogentec SA (Seraing, BE).

### Plasmids and recombinant proteins

pGEX-2T-1 with the bovine αGDI was from M Zerial, (Dresden, Germany), PME18S-Flag-TXNL1 (TRP32) from KK Lee (Kyoto, Japan), and pCNA3.1 His-p38a from K Tamura (San Diego, La Jolla, CA). Generation of point mutants was carried out with the Quick-Change Site-Directed Mutagenesis kit (Stratagene, San Diego, La Jolla, CA). Truncated TXNL1 corresponding to the N-terminal (residues 1–122) and C-terminal (residues 105–289) domains were generated by PCR. GST-TXNL1 proteins were expressed in BL21 (Stratagene) cells and purified according to the manufacturer's instructions (Amersham). GST-GDI was produced using BL21(DE3)plysS bacteria (Stratagene) [39]. When indicated, the GST tag was cleaved by Factor Xa, which was then removed using Benzamidine Sepharose 4 fast flow according to the manufacturer's instructions (Amersham).

### In vivo experiments

TXNL1 expression was silenced in HeLa cells by Dicer-generated siRNA duplexes. Briefly, after PCR amplification to generate T7 promoter-based DNA templates, the target sequence (nucleotide 181–760) was transcribed *in vitro* to generate dsRNA. Dycing reaction and purification of d-siRNAs were according to the manufacturer's instructions (Invitrogen, Carlsbad, CA). Cells were transfected with d-siRNA using Lipofectamine 2000 24 h after seeding, and 24 h later they were split and grown for an additional 48 h time period (total time of silencing: 72 h). DNA transfection was performed with either Fugene6 or CaPO_4_ 24 h after seeding and cells were incubated for 36 h.

### In vitro experiments

Rat liver cytosol (RLC) preparation and endosome fractionation by floatation in sucrose gradients were described [39], and performed in the presence of inhibitors of proteases (10 µg/ml aprotinin, 10 µM leupeptin and 1 µM pepstatin) and phosphatases (0.1 mM vanadate and 50 mM glycerol 2-phosphate or 1 µM okadaic acid). RLC was fractionated by (NH_4_)_2_SO_4_ precipitation and gel chromatography on a Superose 12 column using the SMART system [9]. The assay measuring the capture of Rab5 from purified early endosomes by recombinant GDI was described [9], [39]. Briefly, 20 µg purified early endosomes were incubated for 20 min at 30°C in EEB (75 mM K-Acetate, 30 mM HEPES pH 7.4, 5 mM MgCl_2_) containing 0.3 mM ATP, 3 mM GDP, 1 µM GST-GDI and 100 µg cytosol. When indicated, the reaction mixture was supplemented with 500 nM recombinant TXNL1 or TXNL1^C34, 37S^ (after GST removal) that were either pre-reduced with 1 mM DTT (30 min, on ice) or pre-oxidized with 10 mM H_2_O_2_ (15 min, 25°C). The mixture was then adjusted to 40.6% sucrose, transferred to a TLS55 centrifuge tube and overlaid sequentially with 35% sucrose solution, then with HB (8.6%, w/w sucrose in H_2_O). The gradient was centrifuged at 170000×g for 1 h at 4°C. After centrifugation, GST-GDI with bound Rab5 was found in the load of the gradient — well separated from Rab5 still associated with endosomes, which floated to the 35-HB interface. GST-GDI was then recovered onto glutathione-Sepharose beads, which were then resuspended in SDS gel sample buffer and Rab5 bound to GST-GDI was analyzed by SDS-PAGE and western blotting.

For immunopreciptation, Fugene-transfected HeLa cells were lysed in TNE buffer (20 mM Tris pH 7.4, 150 mM NaCl, 1 mM EDTA) containing 10% glycerol, 1% NP-40, and proteases inhibitors. Lysates were pre-cleared on protein-A Sepharose beads, divided and then incubated with or without the indicated antibodies for 2 h, and then for 1 h with protein-A Sepharose beads. Beads were washed with TNE containing glycerol, resuspended in sample buffer and analyzed by SDS-PAGE and western blotting.

In GST pulldowns, pre-cleared RLC was incubated with 60 nM GST fusion proteins for 2 h in HB. When indicated, the recombinant proteins were either pre-reduced with 1 mM DTT (30 min, on ice) or pre-oxidized with 10 mM H_2_O_2_ (15 min, 25°C). Complexes were recovered onto glutathione-Sepharose beads, washed with EEB and analyzed by SDS-PAGE and western blotting.

### Other techniques

Activation of p38MAPK by oxidative stress [9], [39], quantification of endocytosis with HRP [39] and immunofluorescence [9] were described. Phospho-p38 was measured using an ELISA kit according to the manufacturer's instructions (Cell Signaling, Beverly, MA). Briefly, BHK cells were homogenized in the presence of 10 µg/ml aprotinin, 10 µM leupeptin, 1 µM pepstatin, 1 mM Vanadate and 1 mM ß-Glycerophosphate. After fractionation by flotation in a sucrose step gradient, 5 µg early endosomes were lysed in 1% TX100 and used in the assay. Insulin disulfide reduction assay was performed essentially as described in [19], [40]. Briefly, 0.13 mM bovine insulin were mixed with 5 µM recombinant protein in 0.1 M potassium phosphate pH 7.0, 2 mM EDTA. The reaction was started by adding 2.5 mM DTT and insulin reduction was monitored by recording the absorbance at 600 nm at RT. The nonenzymatic reduction of insulin by DTT was recorded as a control. TXNL1 was identified by tandem mass spectrometry [9] (peptide sequences: GYMDLMPFINK, IDQYQGADAVGLEEK, FQNVNSVTIFVQSNQGEEETTR).
